# The Dynamic Partnership of Polycomb and Trithorax in Brain Development and Diseases

**DOI:** 10.3390/epigenomes3030017

**Published:** 2019-08-21

**Authors:** Janise N. Kuehner, Bing Yao

**Affiliations:** Department of Human Genetics, Emory University School of Medicine, Atlanta, GA 30322, USA; janise.nichole.unger@emory.edu

**Keywords:** epigenetics, polycomb, trithorax, brain development, neurodegeneration, Alzheimer’s Disease, Huntington’s Disease, Parkinson’s Disease

## Abstract

Epigenetic mechanisms, including DNA and histone modifications, are pivotal for normal brain development and functions by modulating spatial and temporal gene expression. Dysregulation of the epigenetic machinery can serve as a causal role in numerous brain disorders. Proper mammalian brain development and functions depend on the precise expression of neuronal-specific genes, transcription factors and epigenetic modifications. Antagonistic polycomb and trithorax proteins form multimeric complexes and play important roles in these processes by epigenetically controlling gene repression or activation through various molecular mechanisms. Aberrant expression or disruption of either protein group can contribute to neurodegenerative diseases. This review focus on the current progress of Polycomb and Trithorax complexes in brain development and disease, and provides a future outlook of the field.

## 1. Introduction

Originally discovered in *Drosophila melanogaster* as antagonistic regulators of the developmental *Hox* genes [1], Polycomb and Trithorax proteins have taken center stage in being some of the most dynamic multimeric complexes involved in development. As organisms advance through development, stem cells progressively lose their pluripotent potential in part because of chromatin reorganization. Cells acquire and maintain tissue and cell type-specific genetic patterns, promoting their differentiation and development of all the body systems. Polycomb group (PcG) and trithorax group (TrxG) proteins regulate the surrounding chromatin environment by co-occupying the same genomic regions [2]. Demonstrating the absolute necessity PcGs and TrxGs for proper development, deletion of some of these proteins results in embryonic lethality, as observed in mouse models [3]. Recently, the importance of the delicate balance between PcG and TrxG proteins throughout brain development—particularly embryonic and adult neurogenesis, aging, neuroprotection and neurodegeneration—is being recognized [4,5,6]. The aberrant expression of either PcG or TrxG proteins is beginning to be associated with neurodegenerative disease, such as Alzheimer’s, Huntington’s and Parkinson’s disease [7,8,9]. The current challenge lies in determining the extent to which PcG and TrxG proteins play in the development and progression of these diseases, and if unique complexes exist that could act as early detection biomarkers.

## 2. Polycomb Group Proteins

Polycomb group (PcG) and trithorax group (TrxG) proteins exhibit profound conservation between plants, worms and mammals [10,11], indicative of their indispensable functions. The antagonistic relationship between PcG and TrxG proteins adds dimension to the epigenome, creating a dualistic epigenetic switch allowing genes to cycle between activation, inactivation, reactivation and even an intermediate state. PcG and TrxG are best known for their roles in regulating gene expression by forming large, multimeric complexes that maintain the local chromatin environment in either a repressed or active state, respectively [12,13]. For example, PcG and TrxG complexes dynamically modulate genes critical for development and cellular differentiation pathways [14]. In the following sections, we discuss the core and accessory proteins that make up these complexes, as well as their roles in the central nervous system (CNS).

### 2.1. Polycomb Repressive Complex 2

**Figure 1:**
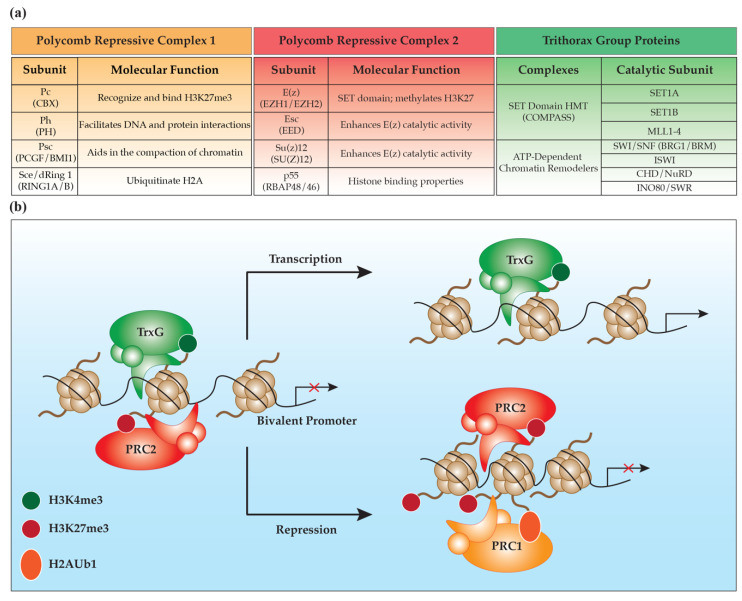
Polycomb and trithorax group protein subunits and complexes: (**a**) polycomb repressive complex 1 (PRC1) and polycomb repressive complex 2 (PRC2) are multimeric complexes composed of core proteins that are responsible for their catalytic activity. Trithorax group (TrxG) complexes: there are six COMPASS-like SET domain histone methyltransferases (HMTs) and four ATP-dependent chromatin remodelers in mammals; arrow with red cross: no transcription (**b**) Embryonic stem cells give rise to differentiated cells over the course of development. Part of this differentiation process requires that bivalent promoters commit to either a repressed or active state. Abbreviations: Pc: polycomb, CBX: Chromobox homolog, Ph: polyhomeotic, PH: polyhomeotic, Psc: posterior sex combs, PCGF: polycomb group finger, BMI1: polycomb complex protein BMI1, Sce/dRing 1: sex combs extra/ ring finger protein 1, RING1A/B: ring finger protein 1A or ring finger protein 1B, E(z): enhancer of zeste protein, EZH1/EZH2: enhancer of zeste homolog 1 and 2, Esc: extra sex combs protein, EED: embryonic ectoderm development, Su(z)12: suppressor of zeste 12, SU(Z)12: suppressor of zeste 12, p55: histone binding protein, RBAP48 and RBAP46: histone binding proteins, COMPASS: Complex of Proteins Associated with Set1, SET: Sur3-9 Enhancer-of-zeste and Trithorax, MLL1-4: mixed lineage leukemia 1–4, SWI/SNF: switch/sucrose non-fermentable complexes, ISWI: imitation switch complexes, CHD/NuRD: the chromodomain helicase DNA-binding/nucleosome remodeling deacetylase complexes, INO80/SWR: inositol auxotroph 80 complexes, BRG1/BRM: BRG1/brahma (BRM)-associated factor (BAF) complexes.

Two multimeric protein complexes comprise the PcG proteins: polycomb repressive complex 1 (PRC1) and polycomb repressive complex 2 (PRC2), collectively working together to generate a repressive chromatin environment [15] (Figure 1A). The basic function of each complex is attributed to the core proteins found in all PRC1s or PRC2s. There are four *Drosophila* core proteins for PRC2: enhancer of zeste (E(z)) protein, extra sex combs (Esc) protein, suppressor of zeste 12 (Su(z)12) and the histone binding protein p55 [16,17,18]. Each *Drosophila* core protein has one or more conserved homologs making up the mammalian PRC2, including enhancer of zeste homologs 1 and 2 (EZH1 and EZH2), embryonic ectoderm development (EED), suppressor of zeste 12 (SU(Z)12) and histone binding proteins RBAP48 and RBAP46. The lysine methyltransferase activity of PRC2, specifically on histone 3 at lysine 27 (H3K27), depends on the catalytic activity of the SET domain within either EZH1 or EZH2 [19] (Figure 1B). For E(z) to have complete catalytic activity to generate the H3K27me3 mark, E(z) requires the presence of both Esc and Su(z)12 [20]. Interestingly, mutations in EZH2, EED and SU(z)12 have all been found in patients with Weaver syndrome, a rare congenital disorder characterized by intellectual disabilities, accelerated bone age and general overgrowth of the head and facial features [21,22,23]. In vitro assays demonstrate that several of the identified mutations in these core proteins reduce the histone methyltransferase ability of PRC2 [24,25].

How mammalian PRC2 is recruited to chromatin is not well understood. In *Drosophila*, there are DNA sequences called polycomb response elements (PREs) that recruit PcG proteins; however, PREs are not conserved between fly and mammals [26]. For example, the *Drosophila* polycomb proteins pleiohomeotic (Pho) and pleiohomeotic-like (Pho-l) contain a DNA binding domain and interact with PcG proteins [27]. However, the mammalian homolog of Pho—YY1—does not colocalize with PRC2 in mammalian embryonic stem cells (ESCs) [28], suggesting that YY1 does not recruit PRC2 to DNA in mammals. In 2009 and 2010, the first promising PREs were identified in mammals, PRE-*kr* in the mouse and D11.12 (a 1.8 kb region between *HOXD11* and *HOXD12*) in human cells [29,30,31]. PRE-*kr* regulates the *MafB/Kreisler* gene’s spatial expression during hindbrain formation in mouse and flies by stably recruiting PRC1 in the anterior and TrxG proteins in the posterior hindbrain [29]. D11.12 was discovered in human ESCs based on its similar properties to *Drosophila* PREs, such as nuclease sensitivity and YY1 binding sites within GC-rich regions [30,31]. The enigmatic quest to identify and characterize PREs in the human genome remains a challenge due to the likelihood that PRC2 protein recruitment depends on multiple components and not just a sequence motif. Furthermore, mammalian PRC1 and PRC2 have multiple isoforms of their subunits, allowing them to function at different developmental stages and at different genetic loci, further complicating the understanding of PREs in mammals [3]. One such example is the mammalian versions of E(z), EZH1 and EZH2. PRC2 complexes that contain EZH2 are most commonly found in embryonic stem cells and in highly proliferative cells. On the other hand, those with EZH1 are predominately found in differentiated and non-dividing cells [32].

The function of PRC2 depends on the numerous combinations of cofactors that associate with it, such as AEBP2, JmjC-domain proteins and chromodomain helicase DNA-binding protein 5 (CHD5) [33], enhancing its ability to function spatiotemporally. Immunoprecipitation experiments repeatedly show that the zinc finger, AEBP2, frequently associates with PRC2 and is required to optimize the methyltransferase activity of PRC2 [34,35]. Evidence suggests that PRC2 complexes containing AEBP2 can promote cell migration and contribute to neural crest development [36,37]. The JmjC-domain protein, Jmjd3, specifically removes H3K27me3 marks at genes highly expressed in neural progenitor cells (NPCs), such as *Pax6*, *Nestin* and *Sox1*, and is required for neural lineage commitment [38]. CHD5 is a neuron-specific chromatin remodeler that is required for terminal neuronal differentiation [39]. Small hairpin knockdown of *CHD5* in SH-SY5Y cells mitigate the activation of critical neuronal genes such as *PHOX2A*, *RARA* and *TBX2*. Interestingly, the chromodomain of CHD5 binds to H3K27me3 modifications and assists PRC2 in depositing more H3K27me3 marks to maintain the repression of PRC2 target genes during adult neurogenesis.

### 2.2. Polycomb Repressive Complex 1

PRC1 is also composed of a set of four core proteins, including polycomb (Pc), polyhomeotic (Ph), posterior sex combs (Psc) and sex combs extra/ring finger protein 1 (Sce/dRing 1) [15] (Figure 1A). In vertebrates, each of the core proteins has numerous paralogs that can have either redundant or unique functions [26]. The chromodomain in Pc is responsible for recognizing and binding H3K27me3, facilitating the recruitment of PRC1 to induce structural changes in the chromatin [19,40,41] (Figure 1B). There are five mammalian isoforms of Pc (Chromobox Homologs: CBX2, CBX4, CBX6, CBX7 and CBX8) that bind H3K27me3 [42], as well as other methylated histones, non-PcG proteins and even RNA [43,44]. Notably, Cbx4 (Pc2) is unique among the five isoforms, as it is the only one involved in sumoylation [45]. The many combinations of core protein isoforms comprising PRC1 can affect where in the genome different PRC1 complexes bind. Interestingly, different CBX proteins bind to nucleosomes in distinct subnuclear regions [42]. 

The RING1A/B proteins in PRC1 are responsible for the monoubiquitination of lysines on histone H2A, especially at lysine 119 to generate the H2AK119ub1 mark [46,47] (Figure 1B). Knockout of just Ring1B is sufficient to deplete global H2A ubiquitination in ESCs; however, knockout of both Ring1A/B is required to deplete H2A ubiquitination on the inactive X chromosome [46]. H2AK119ub1 can be regulated in an H3K27me3-independent mechanism. Tavares et al. identified two distinct PRC1 complexes in mouse ESCs composed of different PRC1 catalytic subunits and the protein RING1 and YY1 Binding Protein (RYBP) [48]. The first complex, CBX-PRC1, is recruited in the canonical fashion by H3K27me3 interactions; however, the second complex, RYBP-PRC1, is recruited by an unknown mechanism independent of H3K27me3. Knockdown experiments of RYBP show a significant loss of H2AK119ub1 levels, demonstrating the critical role of RYBP in maintaining H2AK119ub1 levels. Furthermore, proteomic and genomic studies have distinguished six PRC1 complex groups that are composed of unique subunits that localize to different genomic regions [49]. All six PRC1 complexes contain a RING1A/B protein and one of the six human Psc homologs, the polycomb group finger (PCGF) subunits. Notably, each unique PRC1 complex has minimal overlapping binding patterns. The remarkable diversity found in PRC1 composition and its variance in genomic localization expands the possible biological function of PRC1 further than the field initially expected. 

## 3. Trithorax Group Proteins

TrxG complexes deposit histone 3 lysine 4 trimethyl (H3K4me3)-activating histone marks as opposed to the H3K27me3 repressive histone marks deposited by PcG complexes. TrxG proteins modify histones to remodel and bind chromatin to promote an active environment. Similar to the polycomb proteins, trithorax proteins are evolutionarily conserved and function in large multimeric protein complexes. In general, the function and localization of trithorax protein complexes are influenced by the binding targets of accessory proteins [50]. We will briefly discuss the three different groups of TrxG proteins, as they have been thoroughly reviewed elsewhere [51].

### 3.1. Trithorax SET Domain Histone Methyltransferases

Initially discovered in yeast, mono-, di- and tri- methylation on H3K4 is catalyzed by a complex of proteins associated with Set1 or Complex of Proteins Associated with Set1 (COMPASS) [52,53] (Figure 1A,B). There are six COMPASS-like complexes in mammals comprised of one of the following catalytic domains: SET1A, SET1B or Mixed Lineage Leukemia 1–4 (MLL1–4) [54,55]. In addition to their catalytic subunit, COMPASS complexes are also composed of a core group of proteins: WD Repeat Domain 5 (WDR5), ASH2, Retinoblastoma-Binding Protein 5 (RBBP5) and DPY30 [56,57]. As a complex, these proteins are responsible for most of the H3K4me3 present globally, and thus global gene activation [54,55]. Lentiviral infection of shRNAs targeting either *Dpy30* or *RbBP5* in mouse ESCs results in a significant reduction in H3K4me3, causing defects in ESC differentiation into neuronal lineages, but not self-proliferation [54]. The MLL complexes are best known for their proto-oncogenic roles in leukemia; however, mutations in these genes also cause neurodevelopmental disorders such as Kabuki syndrome and Wiedemann–Steiner syndrome. Kabuki syndrome is a rare disorder characterized by numerous skeletal deformities and moderate intellectual disabilities [58]. Interestingly, in a screening of 110 families with Kabuki syndrome, 74% had frame shift mutations in *MLL2*, resulting in haploinsufficiency. Whole exome sequencing identified de novo mutations in *MLL1* in individuals diagnosed with Wiedemann–Steiner syndrome, another extremely rare neurodevelopmental disease [59]. Like polycomb proteins, the function of trithorax proteins vary based on what accessory proteins they are in contact with, permitting their spatiotemporal regulation. For example, the zinc finger protein ZNF335 (a known causal gene for microcephaly and essential for NPC self-renewal, neurogenesis and neural differentiation) is a component of a COMPASS-like complex containing MLL and SETD1A [60]. Together, this complex modulates the master neural gene regulator RE1-Silencing Transcription Factor/Neuron-Restrictive Silencer Factor (REST/NRSF), which promotes NPC proliferation by inhibiting genes required for neural differentiation [61].

### 3.2. ATP-Dependent Chromatin Remodelers

As their name implies, ATP-dependent chromatin remodelers use energy derived from ATP to physically slide/evict nucleosomes and rearrange the chromatin environment. The ability of these proteins to remodel the chromatin is modulated by histone post-translational modifications, histone variants and the length of the linker DNA sequence between two nucleosomes [62]. Trithorax ATP-dependent chromatin remodelers are categorized into four groups based on their ATPase domains: the switch/sucrose non-fermentable (SWI/SNF) complexes, the imitation switch (ISWI) complexes, the chromodomain helicase DNA-binding/nucleosome remodeling deacetylase (CHD/NuRD) complexes and the inositol auxotroph 80 (INO80/SWR) complexes [50,63] (Figure 1A). In the mammalian brain, these chromatin remodelers have essential roles in proper brain development and function. For example, in the CNS, the SWI/SNF complexes, also known as BRG1/brahma (BRM)-associated factor (BAF) complexes, exist in a regimented spatiotemporal manner. The transition from ESCs to neural progenitor cells (NPCs) to mature neurons is modulated by the specific BAF complexes esBAF, npBAF and nBAF, respectively [64,65]. Mutations in the core components or accessory proteins of these complexes are increasingly being reported in neurological disorders, such as intellectual disabilities, schizophrenia and microcephaly [66,67]. Additionally, the mammalian ISWI proteins SNF2L and SNF2H (encoded by *SMARCA1* and *SMARCA5*) are also involved in NPC differentiation, as deletions within either gene results in abnormal brain growth and attenuated proliferation [68,69]. Using whole exome sequencing, mutations in *SMARCA1* have been identified in patients with microcephaly, Rett syndrome and schizophrenia [67,70,71]. However, each of these cases are isolated examples, prompting further work to be done in exploring the role of *SMARCA1* in neurological disorders. The *Chd7* gene, the encoding part of the CHD/NuRD complexes, shows specific and dynamic spatiotemporal expression patterning in the developing mouse brain, and is essential during neuronal differentiation [72,73,74,75,76]. Interestingly, de novo mutations that result in haploinsufficiency of *CHD7* are the major contributor in CHARGE syndrome, a rare congenital disease where patients have abnormal brain structure formation [77,78]. Finally, functional roles for the INO80/SWR complexes are the least explored chromatin remodelers in the brain. Recent work suggests several unique roles for INO80 in transcription regulation and DNA replication and repair [79]. In summary, ATP-dependent chromatin remodelers can have very broad or very specific roles in the mammalian brain. 

### 3.3. TrxG Response Elements (TREs)

As with polycomb recruitment, trithorax recruitment in mammals also does not appear to be conserved. In the attempts to determine how TrxG proteins are recruited in the mammalian systems, several hypotheses have been proposed. One such hypothesis is that regions dense in CG dinucleotides, such as CpG islands, could recruit them [28,80,81]. Supporting this, the mammalian versions of the trithorax proteins, MLL1 and MLL2, both have a CXXC domain that recognizes and binds to unmethylated CpG regions, which is not conserved in flies [82]. However, several other publications found conflicting evidence, where CG density failed to predict or was not sufficient to recruit polycomb and trithorax response elements [29,31]. These experiments only analyzed a single locus compared to the genome-wide approach taken in the previous studies, suggesting that other mechanisms in addition to CG content may be required for polycomb and trithorax recruitment in mammals. Recent work paired bioinformatics with the reporter luciferase assay to predict candidate PREs and TREs in the human genome [83]. Five putative PREs and four TREs characterized by either enrichment of H3K27me3 and strong signals for EZH2 and EED, or enrichment of H3K4me3 and strong signals for MLL1 and WDR5, respectively, were identified. Notably, CG content was also not found to be directly coupled to PcG or TrxG proteins; however, CpG islands appeared to correlate more strongly with TREs compared to PREs. Another hypothesis suggests that noncoding RNA molecules, such as long noncoding RNAs (lncRNAs), could have recruitment function. For example, the lncRNA *HOTTIP* activates *HOXA* genes by directly targeting the WDR5/MLL complex, allowing for H3K4me3 marks to be dispersed [84]. Furthermore, *HOTAIR* acts as a scaffold to bring PRC2 and the H3K4 demethylase LSD1 into close enough proximity to help resolve bivalent promoters to retain H3K27me3 marks [85]. The most recent hypothesis proposes that DNA modifications may be important for PcG and TrxG target recognition. In flies, the repressive DNA modification, *N*^6^-methyladenosine (6mA), epigenetically regulates genes involved in neurodevelopment and neuronal functions by recruiting PcG and TrxG proteins [86]. Co-immunoprecipitation experiments determined that the demethylase for 6mA, DMAD, interacts with the TrxG protein Wds (WDR5 in mammals) to coordinately remove 6mA marks, promoting an active chromatin environment. Conversely, depletion of DMAD results in the failure of Wds recruitment, an accumulation in 6mA and consequently recruitment of the PcG to repress these regions. Likewise, in mammals, 6mA expression is correlated with an increase in PRC1 ubiquitination of H2A and to a lesser extent H3K27me3, preserving silenced gene regions [87]. Both pieces of evidence suggest that 6mA is correlated with PcG binding and could potentially serve as an interesting mechanism for PcG–TrxG commitment. Overall, there are many proposed mechanisms that could contribute to polycomb and trithorax recruitment in mammals. Individually, each hypothesis may be supported by individual situations; however, it is far more likely that these mechanisms act cooperatively to recruit polycomb and trithorax complexes.

## 4. PcG and TrxG Proteins in the CNS

From the above discussion, it is evident that polycomb and trithorax complexes have numerous roles in development that are influenced by the co-factors with which they interact. Since identifying these various accessory proteins, researchers have begun elucidating their copious functions in the nervous system. In the following sections, we discuss the functional roles of Polycomb and Trithorax complexes in a variety of neural processes, including neurogenesis, CNS development, gliogenesis and neuronal migration. 

Mammalian neurogenesis is the process by which NPCs differentiate to form new neurons, and have been thoroughly reviewed elsewhere [88]. This process occurs during embryonic development and in several regions of the adult brain: the subventricular zone of the lateral ventricles and subgranular zones of the dentate gyrus [89]. NPCs generate fate-restricted radial glia cells that give rise to intermediate progenitor cells, neurons, astrocytes and oligodendrocytes, which then migrate outward to form the outer layers of the brain [90]. Part of this differentiation process requires that bivalent domains, silenced regions that simultaneously contain H3K27me3 and H3K4me3 marks, in ESCs to commit to either a repressed or active state (Figure 1B). These regions are considered “poised” because of their potential to be activated. In mouse ESCs, many bivalent domains are found near transcription start sites (TSSs) encoding transcription factor genes critical for proliferation (*Sox*, *Fox*, *Pax*, etc.) [91]. In addition, bivalent domains are also found near genes critical for development (*Pax2* and *Wnt8b*) and neural development (*Fgf8* and *Prok1*). In mice, as ESCs differentiate into either NPCs or mouse embryonic fibroblast (MEF), their bivalent promoters start to commit to either a repressive or active state [92]. As expected, housekeeping genes maintain their H3K4me3 mark in all cell types, but genes specific to either NPCs (*Olig1*, *Neurog1* and *Fabp7*) or MEFs (*Pparg*) lose their H3K27me3 mark, but maintain their H3K4me3 mark or maintain a bivalent state during differentiation. PcG and TrxG proteins undoubtedly have essential roles in the early stages of neurogenesis, as ESCs differentiate into NPCs to initiate the development of the CNS.

### 4.1. PRC1 in Neurogenesis and CNS Development

Mammalian PRC1 complexes are far more diverse compared to PRC2 because each core component has numerous paralogs that have non-overlapping functions. Vogel et al. revealed spatiotemporal expression patterns of PRC1 members during both neurogenesis and brain development using in situ hybridization in mouse brain [93]. In general, PRC1 complexes composed of Ring1a, Rnf2, M33 and Ph2 show the greatest expression in the ventricular zone in highly proliferative cell populations, whereas Cbx4, Cbx7, Bmi1 and Mel18 are more highly expressed in differentiated neurons in the outer layers of the developing brain. All PRC1 complexes contain RING1A/B [49] and knockout studies demonstrate that Ring1B has critical roles in mouse neocortical development [94]. The Ring1B catalytic unit of PRC1 controls the transition from the neurogenic phase to the astrogenic phase in NPCs by suppressing the transcription factor neurogenin 1 (Ngn1). In vertebrates, the Psc proteins are known as polycomb group finger (PCGF) and PCGF4, or Bmi1 [95], and are required for NPCs’ self-renewal by repressing the cyclin-dependent kinase inhibitor genes *p16^Ink4a^*, *p19Arf* and *p21* in mouse [96,97,98]. When *Bmi1* is mutated, there is a significant reduction of NPCs in the subventricular zone (SVZ) of adult mice. PRC1 complexes take on a broad range of specialized functions depending on which core proteins associate with each other. It is surprising that additional work has not yet been done to elucidate novel functions of PRC1 in brain development. Further investigation is warranted to determine the existence of spatiotemporal PRC1 complexes and what unique contributions they could have towards brain development.

### 4.2. PRC2 in Neurogenesis and CNS Development

Members of PRC2 have been implicated in numerous roles including, but not limited to: neuronal identity, proliferation, differentiation and neuronal morphology [99]. Here, we discuss the roles of the core proteins (EZH2, EED, SUZ12 and RBAP46/48) and their unique involvement in neurogenesis. For ESCs to maintain their pluripotent state, PcG proteins target and repress key developmental regulators, such as *Hox* genes and transcription factors such as *Fox* and *Sox* genes, to prevent differentiation [100,101,102,103]. EZH2 is predominately expressed in proliferating cells such as ESCs and NPCs. Several studies have reported that deletion of *Ezh2* in mouse NPCs disrupts the timing and number of neurons during neurogenesis and cortical plate thickness [94,104,105]. Conditional knockout of *Ezh2* in the forebrain or midbrain of mice before the onset of neurogenesis shifts the balance of NPC self-proliferation towards NPC differentiation, and prevents expansion of the cortex [104,105]. Ezh2 also promotes the development of brain region identities, as loss of *Ezh2* in the midbrain results in ectopic expression of forebrain-specific genes *Foxg1* and *Pax6* and repression of midbrain markers *Pax3* and *Pax7* [105]. Interestingly, genes that inhibit cellular proliferation (*Cdkn2a* and *Cdkn2c*) and Wnt signaling (*Wif1* and *Dkk2*) are de-repressed upon *Ezh2* loss. In addition to inappropriate expression of mature neurons, mice null for *Ezh2* have significantly reduced levels of H3K27me3 in both their NPCs and the neurons that are generated from them [104]. Consequently, genes only expressed in differentiated neurons of the cortex, such as *Bcl11b*, *Myt1l*, *Mef2c* and *Neurod6*, are upregulated during early cortical development. Additionally, loss of H3K27me3 profoundly disrupt the regulation of key transcription factors implicated in ventricular zone neurogenesis [106]. Mice with a conditional knockout of *Ezh2* show an upregulation of GABAergic interneuron markers (*Pax2*, *Pax5* and *Pax8*), and a downregulation of Purkinje cell gene markers (*Rora*, *Olig2* and *Olig1*). Consequently, the increase of GABAergic interneurons and decease of Purkinje cells results in an underdeveloped cerebellum early in development. Interestingly, in the subventricular zone, there is a unique population of astrocytes termed neurogenic astrocytes, that retain a “stem cell like” state and continue to produce neurons into adulthood [107]. What distinguishes neurogenic astrocytes from non-neurogenic astrocytes is the robust expression of *Ezh2*. In neurogenic astrocytes, Ezh2 has two distinct functions. The first function is to maintain the self-renewal production of more astrocytes by repressing the cell cycle inhibitor *Ink4a/Arf* or *Cdkn2a*. The second function is to promote neuronal lineage differentiation where neurogenic astrocytes produce neurons via inhibition of transcription factor Olig2. It would be interesting to determine if neurogenesis could be reinstated in the non-neurogenic astrocytes by expressing *Ezh2*, which could be a model for regenerative medicine.

Less work has been done to determine functional roles of EED, SUZ12 and RBAP46/48 in neurodevelopment. A recent study determined that dormant and actively proliferating NSCs in the SVZ express Eed [108]. Eed is required for NSC proliferation and neurogenesis, as conditional deletion of *Eed* results in NSC differentiation and a reduction in new neuronal numbers, respectively. The *Hox* homeotic genes are established targets of PcG that encode transcription factors that help to regulate anterior–posterior body planning [109,110]. Interestingly, in the embryonic CNS of bilateria animals, there is no *Hox* gene expression in the anterior/brain region of the embryo due to PcG repression of these *Hox* genes [110,111]. Mutations or knockouts of the PRC2 protein, Esc in *Drosophila* or Eed in mammals, results in the loss of H3K27me3 marks in the developing CNS [112]. Furthermore, the anterior/brain region of both species exhibit an increase and extended period of NPC proliferation, whereas the posterior/nerve/spinal cord regions are unaffected by PRC2 loss. In addition to repressing *Hox* gene expression in the brain, PRC2 also promotes the expression of transcription factors (Hbn, Rx, Dpn, etc.) unique to the developing embryonic brain [113]. Ectopic expression these brain transcription factors in the *Drosophila* nerve cord or wing disc is sufficient to reprogram the spinal cord to exhibit a more brain/CNS-like structure. Rescue experiments conducted in *Esc* mutant embryos demonstrate that expression of Tll, Erm or Tetra transcription factors can ameliorate the reduced proliferation phenotype [113]. These findings suggest that PRC2 specifically promotes the expansion and development of the anterior/brain regions by repressing *Hox* gene expression in the brain and driving the expression of brain transcription factors [112,113,114]. During early embryonic nervous system development of both mice and humans, Eed and Suz12 proteins are continuously expressed from embryonic days 9–14 in multiple brain regions [115]. Homozygous knockout mice of either *Eed* or *Suz12* do not develop past the gastrulation phase [116,117]; however, mice heterozygous for *Suz12* can survive, but display a wide range of brain and neural tube defects [118]. The fourth core protein, RBAP46/48 is best known for its general role as a histone chaperone that helps to maintain chromosome stability [119]. Very little is known about how RBAP46/48 affects neurogenesis or brain development. One could speculate that even though RBAP46/48 is not required for the enzymatic activity of PRC2 [120], its chromatin remodeling ability could aid in repression of NSC-specific genes by condensing the chromatin during differentiation. 

### 4.3. TrxG in Neurogenesis and CNS Development

Comparatively, much more work has been done to elucidate the molecular mechanisms of PcG functions in neurogenesis and CNS development than TrxG proteins. ATP-dependent chromatin remodeler, CHD7, is known to have essential roles in early cerebellar development and adult neurogenesis. Cre inactivation of *Chd7* at embryonic day 8.5 in mouse granule cells of the cerebellum results in cerebellar differentiation defects and mis-localization of Purkinje cells [73]. During adult neurogenesis, loss of *Chd7* abrogates neuronal differentiation by preventing the remodeling and activation of the promoters *Sox4* and *Sox11*, essential genes for proper neuronal differentiation [74,121]. Furthermore, TrxG member Mll1 is essential for neurogenesis to occur in the SVZ of mouse postnatal brains [122]. Neural stem cells deficient for *Mll1* display normal cell survival, proliferation and glial cell differentiation; however, differentiation into neural lineages is severely hindered. As neural stem cells differentiate, ChIP analysis has revealed that the bivalent promoters of *Mash1*, *Olig2* and *Dlx2* shift so that they become enriched for H3K4me3 activating marks. Interestingly, in cells deficient for *Mll1*, the *Dlx2* promoter retains a strong H3K27me3-repressive mark that spreads further upstream. This suggests that the H3K4me3 catalytic activity of Mll1 regulates *Dlx2* to promote neurogenesis [122].

### 4.4. PcG and TrxG in Astrogliogenesis and Oligodendrogenesis

Aside from neurogenesis, PcG and TrxG proteins are essential for the development of glial cells, such as astrocytes and oligodendrocytes. During early neocortical development (embryonic day 11.5), there is rapid and symmetric division of NSCs, followed by asymmetric NSC division to initiate neurogenesis [123,124]. By late embryonic development (embryonic day 17.5), neurogenesis concludes and switches to gliogenesis, as reviewed elsewhere [5]. During the termination of neurogenesis, both PRC1 and PRC2 are necessary to promote astrogliogenesis [94]. In fact, deletion of the core subunits *Ring1B*, *Ezh2*, or *Eed* in mouse neocortical NPCs prolong neurogenesis and delay entry into the astrogenic phase. Notably, PcG proteins repress the transcription factor neurogenin 1 (Ngn1), a known suppressor of astrogliogenesis [125]. Silencing of *Ngn1* is a key prerequisite in the transition of NPCs to glial fate. An interesting observation is that as expected—when NPCs differentiate into neurons and astrocytes, Ezh2 decreases; however, the NPCs that differentiate into oligodendrocyte-lineage retain high levels of Ezh2 from precursors to mature oligodendrocytes (OLs) [126]. When *Ezh2* is overexpressed in embryonic day 14 mouse NSCs, there is an overproduction of OLs and a reduction in astrocyte production. The dynamic epigenetic regulation involved in oligodendrogenesis has been recently reviewed [127]. The SWI/SNF member Brg1 is necessary and sufficient for oligodendrogenesis [128]. Interestingly, Brg1 is directed to oligodendrocyte-specific enhancer elements by the transcription factor Olig2, thus promoting OL lineage differentiation and subsequently CNS myelination. Furthermore, Brg1 and Olig2 directly target the gene that encodes Chd7, promoting its activation [76]. Chd7 co-immunoprecipitation identified Sox10, a critical transcription factor in the development of OLs, to associate together and functionally regulate myelination and remyelination of the CNS. 

### 4.5. PcG and TrxG in Neuronal Migration

The majority of studies have focused on the involvement of polycomb and trithorax proteins in the neurogenic aspect of brain development. Another key aspect of proper brain development is the migration of neuronal cells to the outermost layers of the brain. In the developing mouse hindbrain, Ezh2 controls dorsal–ventral pontine neuron migration by regulating netrin 1 (an axon guidance molecule) and restricting various *Hox* gene expressions through the anterior extramural stream (AES) [129,130]. Similar to how the BAF complexes switch out subunits during NPC differentiation, the CHD/NuRD complex also exchanges the CHD ATPase subunit during mouse cortical development [131]. Deletions of *Chd3*, *Chd4* or *Chd5*, followed by rescue experiments, demonstrate the non-overlapping functions of each CHD/NuRD complex. Subsequently, CHD4-containing complexes promote NPC proliferation in the deep cortical layers. CHD5 complexes regulate early neural migration, and CHD3 complexes facilitate late migration, as well as the establishment of features that distinguish mature neurons. Additionally, the brahma (Brm) subunit of the BAF complexes is critical for cortical neuron migration in mice, as *Brm^−/−^* mice have less neurons that reach the cortical plate relative to wild type mice [132]. *Brm* expression is orchestrated by the histone deacetylase HDAC2 when it is nitrosylated. Nitric oxide (NO) acts as an external stimulus that affects the localization, interaction and function of proteins that it modifies (S-nitrosylation), such as HDAC2 [133]. Importantly, NO signaling is critical in regulating NPC proliferation and adult neurogenesis [134], as well as cerebellar cell migration [135]. Furthermore, when neuronal nitric oxide synthase (nNOS) is embryonically deleted in mice, cortical migration is disrupted [132].

### 4.6. PcG and TrxG in Neuroprotection and Aging

Brain injury induced by impaired blood flow, otherwise known as ischemia or stroke, is the second leading cause of death in the United States [136]. A neuroprotective response to ischemia occurs when the brain has been subjected to brief, non-damaging ischemic events to build a protective tolerance known as ischemic tolerance [137]. Studies have shown that ischemic tolerant brains have an increased abundance of PcG proteins, particularly SCHM1, BMI1 and RING2 (members of PRC1), as well as repressor histone variants H2A and H2B [138]. PcG proteins bind at the promoters of *Kcna5* and *Kchab2* (two potassium ion channels in the brain known to be down regulated in ischemic tolerant brains), repressing them, and essentially protecting neural cells from injury. Interestingly, potassium channel blockers improve stroke recovery in animals as well as protect neurons from apoptosis [139,140]. Targeting polycomb complexes to increase their abundance after a stroke has recently been proposed as a putative treatment option [141]. The involvement of polycomb proteins in aging and neural stem cell proliferation are also thought to protect and promote recovery of the brain after an ischemic injury [141].

Neurodegeneration is an inevitable part of aging, whose molecular mechanism is poorly understood. Experiments performed in *Drosophila* have begun to elucidate this mechanism. Flies that are heterozygous for mutations in either *E(z)* or *Esc* have a longer life span, are resistant to oxidative stress and have reduced levels of H3K27me3 [6]. Antagonistically, when flies are heterozygous for *Trx*, the opposite phenotypes are observed, suggesting that lifespan and oxidative resistance are dependent on H3K27me3 levels. Notably, genome-wide mapping of polycomb proteins have failed to target canonical genes associated with longevity, such as *Sir2*, *Rpd3*, *Foxo* and *InR*. However, a putative target of E(z), *Odc1*, is involved in the enzymatic processing of polyamines, which have been linked to oxidative stress resistance [142] and increased lifespan [143]. 

During aging, there is an imbalance of molecular damage caused, in part, by reactive oxygen species (ROS) that is combated by antioxidant defenses [144]. Most intracellular ROS are generated by the mitochondria [145,146,147]. Mouse studies demonstrate that the polycomb group protein, Bmi1, modulates aging by repressing p53 signaling to promote neuron survival and antioxidant defenses [144]. The function of p53 in aging and cellular oxidative metabolism is ambiguous, as p53 has both pro- and anti-aging/oxidant properties in proliferative cells [148,149,150,151]. Mice deficient for *Bmi1* show signs of premature aging (e.g., presence of cataracts in the eyes) and have shorter lifespans compared to wild type littermates [144]. In addition, *Brm^−/−^* neurons have augmented apoptosis due to neurotoxic reagents, such as camptothecin, β-amyloid plaques and ROS. ChIP experiments demonstrate enrichment of p53 at the promoters of antioxidant defense genes, such as *xCT* and *Sod2*, in *Brm^−/−^* neurons. This evidence suggests that, in the context of postmitotic neurons in the adult CNS, p53 has both pro-aging and pro-oxidant properties.

## 5. PcG and TrxG in Neurodegenerative Diseases

As described in the sections above, polycomb and trithorax proteins are essential during brain development and are responsible for establishing early developmental features that are carried into the adult brain. Maintenance of proper chromatin environments to control gene expression is critical to neuron function and survival. Recent work has made apparent the critical role of PcG and TrxG proteins in preventing neurodegeneration. When PRC2 is conditionally depleted in adult mouse projection neurons, there is ectopic de-repression of genes encoding transcriptional regulators of non-neural (*Pax6* and *Tbx20*), non-projection neuron (*Eomes* and *Pou4f1*) and death-promoting genes (*Pmaip1*, *Cdkn2a/b* and *Igfbp3*) [152]. Furthermore, neurons with increased expression of death-promoting genes exhibit a known neurodegenerative phenotype known as dark cell degeneration, demonstrating the importance of PRC2 to suppress neurodegeneration. In the following sections, we discuss known roles of PcG and TrxG proteins in various neurodegenerative diseases.

### 5.1. Huntington’s Disease

Huntington’s Disease (HD) is an autosomal dominant, genetic disease caused by a 40 or more trinucleotide repeat expansion (CAG) in the *huntingtin* gene (*HTT*) [153]. The age of onset is typically in the 40s and individuals with this disease suffer from motor, cognitive and psychiatric symptoms [154]. Work in the field has uncovered putative roles of PcG and TrxG proteins and how they may contribute to HD. 

Before we can understand the dynamic interaction of PcG proteins with mutant *HTT*, it is important to understand the essential molecular interactions PcG proteins have with wildtype *HTT*. PcG proteins were initially thought to interact with Htt as mouse embryos null for either Htt or PRC2 displayed similar phenotypes (i.e., failure to repress growth factors, *Nodal* and *Fgf8* and transcription factors, *Evx1*) [155]. When comparing wild type *Htt* to null *Htt* in embryonic day 7.5 mouse embryos, the null embryos show ectopic expression of *Hoxb1*, *Hoxb2* and *Hoxb9*, all of which are regulated by PRC2 [156]. This finding suggests that a normal function of Htt could be to facilitate PRC2’s repressive function of Hox genes during development. Co-IP experiments on day 4 embryoid bodies (EBs) generated from either wild type or null *Htt* animals demonstrate that wild type Htt associates with Ezh2 and Suz12 [156]. Also, ChIP experiments on these EBs show that only full length Htt protein and H3K27me3 marks are at the *Hoxb9* sequence. When comparing wild type Htt to mutant Htt, both proteins stimulate PRC2 activity; however, more PRC2 activity correlate with increasing polyglutamine length in mutant Htt. The role that PRC2’s association with mutant *Htt* might play in the pathology of HD remains to be explored. ESCs null for *Htt*, show a loss of H3K27me3, specifically at bivalent promoters destined have H3K4me3 enrichment in NPCs [157]. This is a startling finding because it suggests that a normal biological function of huntingtin is to remove H3K27me3 marks. Whereas null Htt is mainly associated with loss of H3K27me3, mutant Htt mainly affects H3K4me3 levels. For example, in Htt mutant NPCs, the vast majority of TSSs show decreasing levels of H3K4me3, which is correlated with decreased transcription at these sites [157]. Similar results were found in human HD postmortem brains, where of the 720 genes identified with differential H3K4me3 levels, 616 genes had reduced H3K4me3 in HD brains relative to control healthy brains [158].

Recent studies have started to explore how non-protein coding genes, such as long non-coding RNAs (lncRNAs), contribute to HD, which has been discussed elsewhere [159]. Several studies demonstrate that lncRNAs interact with PRC2 and sequester it to their target genes, although the mechanism behind this is unknown [160,161]. For example, the lncRNA *HOTAIR* is required for PRC2 to deposit H3K27me3 marks throughout the *HOXD* locus for its repression [161]. One possible reason for the absence of PREs in the mammalian genome is that PRCs are brought to target genes by various noncoding RNAs. Microarrays have identified seven lncRNA that are dysregulated in HD brains compared to healthy brains [159]. Of the seven lncRNA, *TUG1* and *MEG3* have previously been found to associate with PRC2 [160,162]. To what end the interaction of lncRNAs with PRC2 has in HD pathogenesis remains to be discovered. 

### 5.2. Alzheimer’s Disease

Alzheimer’s disease (AD) is considered one of the most common neurodegenerative diseases worldwide, and is characterized by memory loss and impairments in cognitive function [163]. These phenotypes are accompanied by β-amyloid plaques, phosphorylated Tau and neurofibrillary tangles that accumulate in the brain [164]. Typically, onset does not occur until 60 years of age and is strongly associated with the risk gene *apolipoprotein-E* (*APOE*) ε4 [165]. However, there is a rare early onset form known as familial AD that is linked to mutations in the key AD risk genes *amyloid beta precursor* (*APP*), *presenilin 1* (*PSEN1*) or *presenilin 2* (*PSEN2*) [166]. Research has mainly focused on risk gene discovery, cognitive function, neurodegeneration pathologies and epigenetic modifications. The dynamics of the chromatin environment and the extent to which chromatin remodelers participate in AD are poorly characterized.

There are a handful of studies that observe some association of aberrant PcG and TrxG protein regulation in AD. For example, both lysine methyltransferases Kmt2a and Kmt2b (Mll1 and Mll2, respectively) are involved in memory formation [167], which is impaired in AD patients. In mouse hippocampal neurons, loss of *Kmt2a* partially recapitulates a down-regulated gene list similar to that observed in the mouse AD model. Another study identified that deficiency of PRC1 components responsible for the monoubiquitnation of H2A, Bmi1/Ring1, are associated with late-onset AD [7]. This study observed that, in AD brains and induced pluripotent stem cell-differentiated neurons derived from late-onset individuals, *Bmi1* is silenced. Notably, *Bmi1* silencing is not seen in brains of familial AD patients or other dementia-like diseases. Finally, loss of the brain-specific, ATPase chromatin remodeler CHD5 in primary neurons augments genes with known roles in aging and AD [168,169]. An interesting area for new research would be to identify more brain-specific, chromatin-remodeling proteins and elucidate any roles they could have in neurological diseases. Given that most remodelers are ubiquitously expressed, finding remodelers that are brain-specific could lead to the development of biomarkers.

To characterize and compare the human AD epigenome to the mouse AD epigenome, ChIP experiments on various histone marks, including H3K4me3 and H3K27me3, were computationally profiled [170]. Overall, human and mouse AD models show similar peak overlaps, especially at H3K27me3 peaks, demonstrating conservation of the epigenome between mice and humans. Promoters with increased H3K4me3 correspond to immune genes, whereas decreased H3K4me3 promoters correspond to neuronal genes. This work supports the current hypothesis that AD development could be attributed to an immune response provoked from environmental factors (chronic diseases, obesity and type 2 diabetes) experienced during aging, and genetic factors (gene mutations and epigenetic dysregulations) contributing to cognitive impairments [170,171].

### 5.3. Parkinson’s Disease

Parkinson’s Disease (PD) is believed to be caused by dopaminergic neuron death in the substantia nigra [172], a region of the brain that helps control movement, cognition and motivational reward [173]. Following neuron death, α-synuclein containing protein aggregates, known as Lewy bodies [174], begin to form and impair nerve cell communication, and this is thought to spread to healthy neurons [175]. Individuals with PD suffer from tremors and impaired posture and movement [172,176]. Genetic risk factors have been identified, such as mutations and/or duplication events in the *α-synuclein* (*SNCA*) gene [177]. Of all the neurodegenerative diseases discussed, how dysregulation of PcG and TrxG proteins might contribute to PD pathology is the least studied and understood. 

Patients with PD were initially treated with a compound called L-DOPA, a precursor of dopamine that, once across the blood–brain barrier, is easily converted to dopamine [178]. However, chronic treatment with L-DOPA causes dyskinesia or involuntary muscle movements [179,180] due to increased D1 dopamine receptor signaling [181]. Acute treatment of L-DOPA in terminally differentiated neurons, based on a parkinsonism mouse model, increases the presence of the H3K27me3S28 phosphorylation (H3K27me3S28p) double-mark [8]. Interestingly, H3K27me3S28p reduces the binding abilities of PcG proteins, de-repressing target genes such as *Atf3*, *Npas4* and *Hoxa2*. This effect was found to uniquely occur in dopamine-expressing, medium spiny neurons of the striatum, the primary brain region affected in PD [182].

## 6. Future Outlook and Conclusions

Epigenetic regulation of the chromatin environment through polycomb and trithorax proteins requires a delicate balance to promote healthy and proper brain development, and to prevent the development of neurodegenerative diseases. There has been insufficient work done to identify any clear contributing roles of PcG and TrxG proteins in neurodegenerative disorders, despite their clear necessity in brain development. Drug targeting of PcG or TrxG complexes can be challenging because of these complexes’ diverse association with many gene targets; however, this could be beneficial as many neurological disorders consequently arise because of epigenetic disruption of several genes. Additionally, further research is warranted in identifying PcG or TrxG brain/disease-specific complexes that could serve as biomarkers or specific treatment targets. Given that PcG and TrxG have such essential roles early in development, it would be worthwhile to investigate familial cases of neurodegenerative diseases, and to determine if an altered chromatin environment could be detected before disease onset. Such research could initiate the development of preventative treatments.

Increased efforts are being made to better elucidate epigenetic changes involved in stem cell reprogramming to cultivate cell-replacement therapy for neurodegenerative diseases [183]. Before stem cell therapy can become beneficial, understanding how epigenetic mechanisms, such as chromatin remodeling, promote cell fate by restricting cell plasticity is critical. Currently, bone marrow-derived mesenchymal stem cells (BM-MSCs) are one type of stem cell that is being used to treat HD, AD and PD by genetically overexpressing proteins that are downregulated in each disease, such as brain-derived neurotrophic factor (BDNF), nerve growth factor (NGF) and glial-derived neurotrophic factor (GDNF), respectively [184,185,186]. In their undifferentiated state, mesenchymal stem cells (MSCs) have H3K4me3 and H3K27me3 marks on promoters that control lineage-specifications [187]. Depending on which mark persists during differentiation, the downstream lineage of undifferentiated MSCs is affected. Additionally, alterations in histone modifications are associated with the number of passages MSCs undergo in culture. In early passages of MSCs, there is less H3K27me3 at promoters, but with further passaging, promoters start to maintain H3K27me3 and there is an upregulation of PRC2 [188]. This suggests that there could be an optimal time in which cultured stem cells are best fit to be used for cell-replacement therapy. 

The use of in vitro studies on polycomb and trithorax complexes has benefitted the scientific field in that they have permitted the discovery of their core subunits, their direct targets and their molecular function, as discussed in this review. However, one of the most critical roles of PcGs and TrxGs is to control anterior–posterior patterning during embryonic development [189], which cannot be readily studied in an in vitro system as it requires signaling and cell communication, both of which are lost in single cell 2D cultures. 3D culturing of brain organoids could overcome the single cell limitations of 2D culture methods because they have been developed to contain multiple neuronal cell types, including NPCs, mature neurons and microglia [190,191,192,193,194,195]. Up until very recently, organoids did not develop anterior–posterior and dorsal–ventral axes [196]. Cederquist et al. developed an inducible human pluripotent cell line that expresses the sonic hedgehog (SHH) signaling factor [197]. When these cells are embedded into a pole of a developing organoid, upon doxycycline induction, they will express SHH, generating topographically-patterned organoids. Research is underway to generate more sophisticated organoid models that better recapitulate organogenesis. These improvements include developing methods to further drive anterior–posterior and dorsal–ventral axes, as well as vascular systems to improve oxygen and nutrient distribution for better growth [198].

An important area of polycomb and trithorax research not discussed in this review are the critical roles these complexes have in cancer. Altered expression of PcG proteins, such as EZH2, SUZ12 and BMI1, are found in numerous cancer types, such as prostate, breast, liver and neuroblastomas, to name a few [199]. Mutations in TrxG complexes, such as SWI/SNF subunits *SMARCA/B* or COMPASS member MLL, are also found in ovarian cancer, lung cancer and leukemia [200]. In cancer, the polycomb protein BMI1, in cooperation with MYC, promotes tumor development by repressing tumor-suppressor genes, such as *INK4A* and *ARF* [97,201,202,203]. With regards to cancer prognosis, PcG expression levels could serve as predictive marks for cancer severity. For example, overexpression of *EZH2* is highly correlated with a poor prognosis of prostate [204], breast [205], kidney [206] and lung cancer [207]. Given that *EZH2* overexpression is a reliable predictor of various cancer progressions, several small molecule inhibitors have been developed for therapeutic treatments and are currently in clinical trials [208].

In conclusion, we have discussed a spectrum of functions of PcG and TrxG complexes in the mammalian CNS. Further studies are merited to define clear molecular contributions of these diverse and dynamic multimeric complexes in neurodegenerative diseases.
